# Comparative analysis of patellar tendon, achilles tendon and plantar fascia structure in indoor and outdoor football players: a novel cross-sectional pilot study

**DOI:** 10.1038/s41598-024-54403-3

**Published:** 2024-02-16

**Authors:** Carlos Romero-Morales, Álvaro Berzosa-Rojo, Daniel Di Luca-Calabrese, Sergio Vázquez-González, Vanesa Abuín-Porras, Gonzalo Jaén-Crespo, Fernando García-Sanz, Helios Pareja-Galeano

**Affiliations:** 1 Faculty of Sport Sciences, Universidad Europea de Madrid, Villaviciosa de Odón, Madrid, Spain; 2 Clínica CEMTRO, Madrid, Spain; 3 Department of Physical Education, Sport and Human Movement, Universidad Autónoma de Madrid, Madrid, Spain

**Keywords:** Physiology, Anatomy, Risk factors

## Abstract

Different sport modalities were associate with tendon adaptation or even tendon disturbances, such as volleyball, soccer or basketball. Purpose: the aim of the present study was to determine de difference between indoor and outdoor football players on patellar tendon (PT), Achilles tendon (AT), plantar fascia (FP) and Hoffa’s fat pad thickness assessed with ultrasound imaging (USI). A cross-sectional study was developed with a total sample of 30 soccer players divided in two groups: outdoor group (n = 15) and indoor group (n = 15). The thickness of PT, AT, PF and Hoffa’s fat pad has been assessed with USI. Hoffa’s fat pad reported significant differences for the left side between groups (P = 0.026). The rest of variables did not show any significant difference (P < 0.05). The ultrasonography assessment of the thickness of the PT, AT and PF did not show differences between outdoor and indoor football players. Hoffa’s fat pad resulted showed a significant decrease for outdoor soccer players with respect futsal players. Thus, it can be considered that the load stimuli received in both soccer players were not enough to produce structural adaptations in PT, AT and PF tissues.

## Introduction

The tendon is a fibrous connective tissue with high tensile strength that functions to transfer load from the muscle to the bone. Some tendons have an additional function in storing and releasing mechanical load during stretch–shortening activities such as running, jumping, and throwing. This tendon function serves to improve performance and increase the efficiency of human movement. For example, the patellar tendon (PT) or Achilles tendon (AT) stores and releases high levels of energy during jumping or landing^1^. In this context, the PT measures approximately 30 mm in width by 50 mm in length, with a thickness of 5–7 mm^2^ and, the AT reported a thickness about 6–8 mm^3^.

Tendons has been considered as a plastic connective tissue that adapts depending on their load environment. Farnqvist et al. argued that their plasticity is closely linked with transduction mechanisms. The fibroblasts establish connections with the extracellular matrix through integrins, allowing the cells to sense and adjust to mechanical loading^4^. Tendon morphology, such as thickness, cross-sectional area (CSA) or even pennation fiber angle could be modified in response to mechanical load (both due to excess or deficiency). In recently reviewed studies that examined the adaptation of healthy human tendons in response to mechanical stimuli, consistent evidence was found indicating increased stiffness following high-load interventions that induce significant tendon strains.

Different sport modalities were associate with tendon adaptation or even tendon disturbances, such as volleyball, soccer or basketball. In this line, prolonged or repeated exposure to different training exercises or match situations in football could be related with neural and/or morphological adaptations^5^. Within the same sport, there can also be different modalities, for example soccer or futsal. The main differences between soccer (outdoor football) or futsal (indoor football) are as follows: surface (natural or artificial grass for outdoor football while hard indoor court for futsal), field size (38–42 m for soccer while 18–25 for futsal), ball, number of players (and substitutions that can be made during a match), rules, duration and play stiles^6^. Thus features such as performance acceleration, change of direction, jumps and landings, repeated sprints or decelerations could be different between outdoor and indoor football, resulting in differences in the transmission of loads to the entire musculoskeletal system. Therefore, it could generate different connective tissues adaptations directly related with the joints most involved in load transmission (i.e., ankle and knee)^6^.

Several devices, including magnetic resonance or ultrasound imaging (USI), were proposed as reliable and valid methods to assess the soft tissue structures, such as muscle, joints, tendon or fat pads. Hence, USI has been described a non-invasive, relatively unexpensive and quick procedure to evaluate the musculoskeletal structures in static and dynamic condition^7,8^. Previous studies have studied the reliability of USI for the assessing the PT in healthy participants^9,10^, Hoffa’s fat pad^11^, AT or plantar fascia (PF)^12,13^.

Football players who train and play in different environments (i.e., ground surface, training hours, changes of direction, jumps) could generate difference adaptations in the tendons of the main joints such as the knee and ankle in response to different loads stimuli. Therefore, the aim of the present study was to determine de difference between indoor and outdoor football players on PT, AT, FP and Hoffa’s fat pad thickness assessed with USI.

## Methods

### Study design

A cross-sectional study was developed from March to May 2023. The study was designed and performed under the Strengthening the Reporting of Observational Studies in Epidemiology (STROBE) criteria^14^.

### Sample

A total sample of 30 registered football players aged between 18 and 30 years were recruited and divided in 2 groups: (a) outdoor soccer players (n = 15) and (b) indoor soccer players (n = 15). All the football players (a and b groups) performed 3 training session during a week and 1 match on the weekend. The training sessions for both groups have a duration of 90 min. All the participants had been playing soccer for at least 8 years and were registered players. Exclusion criteria for both groups were as follows: any musculoskeletal condition in the lower limb (i.e., fractures, ankle stability or ankle sprains), metabolic disease, ischemic or neurodegenerative diseases, and any previous surgeries.

### Ethics and registry

Before the study commenced, all participants signed an informed consent form. Ethical requirements for human experimentation and Helsinki Declaration were respected^15^. The Research Ethics Committee at the Universidad Europea (Spain) was approved the study.

### Ultrasound imaging procedure

**Figure 1:**
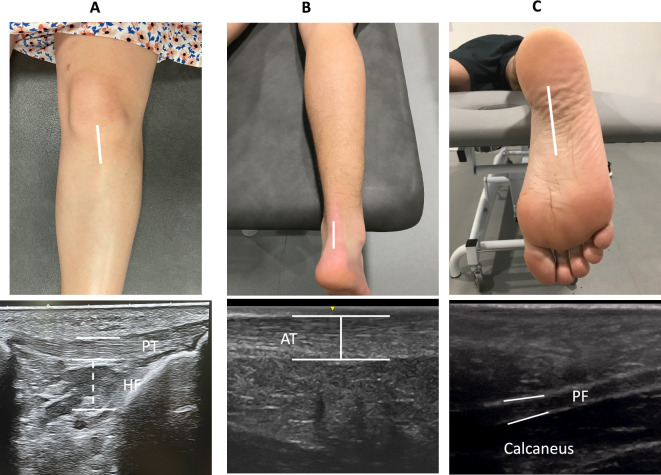
Ultrasound assessment of Hoffa’s pad, achilles tendon and plantar fascia thickness.

All USI measurements were performed by the same therapist (A.B.) with ultrasound imaging specialization. A high-quality ultrasound system (LogiQ P7, GE Healthcare, UK) with a 4–14 MHz linear transducer (L612 RS type with 38-mm footprint) was employed to carried out the B-Mode ultrasound assessments. For the PT and the Hoffa’s fat pads, participants were lying in supine position with both knees flexed at 30° with a pillow placed under the popliteal space during the evaluation. Thickness of the PT has been assessed with the linear transducer placed longitudinally distal to the patella. For the CSA of the PT the probe rotated 90° in the same location. The tendon borders were defined inferiorly by identifying the initial hyperechoic area between the subcutaneous tissue and the deep fascial layer^10,16^. Hoffa's fat pad is located by visualizing the adipose tissue situated directly below the PT (Fig. 1A)^17^. Ultrasound assessment of the AT thickness was performed in prone position with both feet dangling over the end of the table. In this position, AT images were made at 4 cm from the calcaneus with the transducer in a longitudinal view placing the caliper on the upper and lower edges of the tendon (Fig. 1B)^12^. The assessment of the PF thickness was developed in the same position that AT and placing the probe on the line between the medial calcaneal tubercle and the second toe over the PF (Fig. 1C)^13^.

### Data analysis

The statistical analysis was performed by the SPSS package v.22.0 (IBM, Armonk, NY: IBM Corp). First, Shapiro–Wilk was carried out to assess the normality. Second, a descriptive analysis was employed for all the individuals and separately in the two groups. Finally, a comparative analysis between group0 and group1 was performed. Mean, standard deviation (SD) with the Student *t* test and median, interquartile range (IR) with Mann–Whitney U test were carried out for parametric and non-parametric data, respectively. In addition, Levene’s test was employed to assess the equality of variances. Moreover, a multivariate analysis was performed using a linear regression (stepwise selection method; Pin = 0.05; Pout = 0.10) to predict the influence of the dominant leg (right or left) data and group (indoor and outdoor football) on the statistically outcome measurements (showed in the prior described analyses). The dependent variable was the left Hoffa’s fat pad and the independent variables were the group and the dominant leg side (right or left). Additionally, a multivariate predictive analysis used linear regression model performed via stepwise selection method and the R^2^ coefficient to stablish quality adjustments. Significant sociodemographic data variable, age, was used as independent variable.

## Results

Regarding the Table 1, age showed significant differences between groups (P < 0.05), but not show for height, weight and BMI. Considering the Table 2, Hoffa’s fat pad reported significant differences for the left side between groups (P = 0.026). The rest of variables did not show any significant difference (P < 0.05). In this line, PT, AT and PF exhibited a minimal variance between groups with a measured difference of 0.01 cm. Hoffa’s fat pad of the right side reported slightly differences of 0.02 cm with no statistical differences between groups. According to the linear regression analysis, left or right side did not predict this statistically significant difference for the left Hoffa’s fat pad between groups. The lineal regression model showed significant differences (P = 0.048) and R^2^ = 0.137 for PF of the left side. The rest of the dependent variables did not show significant difference.

**Table 1:** Demographic data of the sample.

Data	Outdoor group (n = 15)	Indoor group (n = 15)	*P*-value
Age, years	22.53 ± 1.24	24.26 ± 2.43	0.021
Height, m	1.77 ± 4.89	1.80 ± 6.96	0.215
Weight, kg	73.93 ± 7.44	79.33 ± 14.69	0.149
BMI (kg/m^2^)	23.56 ± 2.13	24.19 ± 3.03	0.518

Data expressed in mean ± standard deviation.

*BMI* body mass index.

**Table 2:** Ultrasound imaging measurements comparison between outdoor and indoor football players.

Measurement	Outdoor football (n = 71)	Indoor football (n = 72)	*P*-value
Right side			
PT-L thickness (cm)	0.40 ± 0.07	0.39 ± 0.04	0.639
PT-T thickness (cm)	0.40 ± 0.07	0.40 ± 0.05	0.847
Hoffa FP thickness (cm)	1.34 ± 0.11	1.36 ± 0.06	0.545
AT-L thickness (cm)	0.44 ± 0.08	0.46 ± 0.06	0.699
AT-T thickness (cm)	0.45 ± 0.07	0.45 ± 0.06	0.979
PF thickness (cm)	0.27 ± 0.04	0.27 ± 0.03	0.929
Left side			
PT-L thickness (cm)	0.37 ± 0.06	0.38 ± 0.04	0.795
PT-T thickness (cm)	0.38 ± 0.04	0.39 ± 0.04	0.629
Hoffa FP thickness (cm)	1.28 ± 0.13	1.38 ± 0.10	0.026
AT-L thickness (cm)	0.45 ± 0.08	0.47 ± 0.07	0.560
AT-T thickness (cm)	0.45 ± 0.07	0.46 ± 0.07	0.653
PF thickness (cm)	0.25 ± 0.04	0.26 ± 0.03	0.926

*PT* patellar tendon, *AT* achilles tendon.

## Discussion

To our knowledge, this cross-sectional study was the first study in the literature to determine the thickness for the PT, AT and PF between outdoor and indoor football players. Regarding the outcome measurements, the USI assessment for the tendinous structures did not report thickness differences. Hence, and based on the results of the present study, it can be observed that no structural changes have occurred in the tendon tissue in response to the different load stimuli to which the two groups of football players are exposed. Authors could postulate that such similar football modalities, these loads differences have not been enough to generate tendon adaptations. Several authors argued that exponential load increases could result in tendon extracellular matrix changes and also tendon morphological parameters (i.e.; thickness or CSA)^18,19^. Thus, regarding the results of the present study authors argued that despite the different players background the load features such as training hours per week, changes of direction or even the substantial difference as the ground surface does not release these adaptation phenomena for the AT, PT and PF structures. However, in musculoskeletal conditions environments, such patellar or Achilles tendinopathy there is a robust literature where authors have been observed structural changes within the tendons and surrounding soft tissue structures. For example, M. Cushman et al. reported that structural changes of the PT were associated with increased development of Achilles tendinopathy and PT conditions such as pain within one year^20^. Based on prior literature is important to note that the clinical significance of the structural tendon findings assessed with USI could be observed in symptomatic or asymptomatic sport populations, being the diagnosis of a tendinopathy condition a clinical entity, not just an imaging evaluation. In this line, the use of doppler ultrasonography could be helpful to the diagnosis of tendon pathology founded a 6.9 increased risk of developing pain and tendon discomfort those participants with presence of neovascularization in tendons^21^. In the same line, Romero et al. reported an increase of AT thickness and CSA at 4 cm and 6 cm from the calcaneus as well as a decrease in Kager’s fat pad length in patients with chronic mid-portion Achilles tendinopathy^12^. In presence of Achilles tendinopathy the thickness of the PF is also reduced compared with healthy controls^13^. Fat pads has been described as adipose tissues that plays a protection role of the joints (i.e.; knee or ankle) helping to distribute forces and reduce friction between different structures^22^. The present study shows differences in the Hoffa’s fat pad that has not been found to be related to the dominant leg, thus it can be concluded that these finding is does not imply as a clinical implication.

An adaptation has been defined as “how an organ or tissue system alters it’s structure or function to best suit it’s environment”^23^. In addition, a tendon adaptation was related to person-level markers (i.e.; sports or load tolerance) or tissue-level changes (i.e.; structural or mechanical properties). Therefore, a tendon tissue under long-term mechanical stimuli could be result in a maladaptive response stressing this structure and being a susceptible source of pathology. Tendons should stay in a “tensional homeostasis” which is a key factor to maintaining the tissue properties^24^. Currently research reported stiffer PT which could be related with the force transmission in elite soccer players, but no AT stiffness differences assessed with a myonotometric tool^25^.

### Limitations and future lines

Several limitations have been observed in the present study. First, the present study just involves healthy participants. Second, dynamic ultrasonography images were not considered. Third, significant differences should be reported for the sociodemographic variable years and should be noted for the results interpretation. Just left and right sides have been compared, not the dominant and no-dominant limbs. Due to the sample size, the results of this pilot study should be interpreted with caution. Similar studies with a larger sample size are needed to achieve statistical power in accordance with this design. Another factor to consider as a limitation could be the small differences in the competition calendar between the two groups and their preparation because of this. The exact years of sports practice were not recorded, this could influence the results. Future studies should be incorporate the doppler ultrasound assessment or the elastography in order to assess the possibility new blood vessels and the tendon stiffness. Moreover, the study of different sports population or upper limb tendons could provide valuable information about the behavior of tendon tissues through an USI examination. Additionally, conducting a study with this design in professional footballers could generate more knowledge about the relationship between load exposure and tendon adaptations in soccer players.

### Clinical relevance

This study support that different football modalities (indoor and outdoor) does not change the tendon structure making some football players more susceptible than others to present a higher injury risk. On the other hand, the present study shows how the ultrasonography for measuring the PT, AT and PF in football players that could be used in clinical assessments.

The present study might could help to coaching teams and athletes in understanding the importance of considering various factors, such as load stimuli, in training and conditioning programs to potentially prevent injuries and enhance the athlete’s performance. Despite no significant different were observed in tendons structure, Hoffa’s fat pad could be affected indicating the need for comprehensive monitoring the biomedicals staffs and the players musculoskeletal health, for example, by a complete assessment based on clinical symptoms, functional test, and ultrasonography.

## Conclusions

The ultrasonography assessment of the thickness of the PT, AT and PF did not show differences between outdoor and indoor football players. Hoffa’s fat pad resulted showed a significant decrease for outdoor soccer players with respect futsal players. Thus, it can be considered that the load stimuli received in both soccer players were not enough to produce structural adaptations in PT, AT and PF tissues.

## Data Availability

The datasets used and/or analysed during the current study available from the corresponding author on reasonable request.
